# Hereditary Spherocytosis due to an *SPTA1* Nonsense Mutation Coinherited With α spectrin^LELY^ in *Trans*

**DOI:** 10.1002/ajh.70021

**Published:** 2025-07-27

**Authors:** María‐Angustias Molina‐Arrebola, Barbara J. Bain

**Affiliations:** ^1^ Unidad de Hematología y Hemoterapia. Área de Biotecnología. Hospital Universitario Poniente Almería Spain; ^2^ Centre for Haematology, St Mary's Hospital Campus of Imperial College London Faculty of Medicine London UK


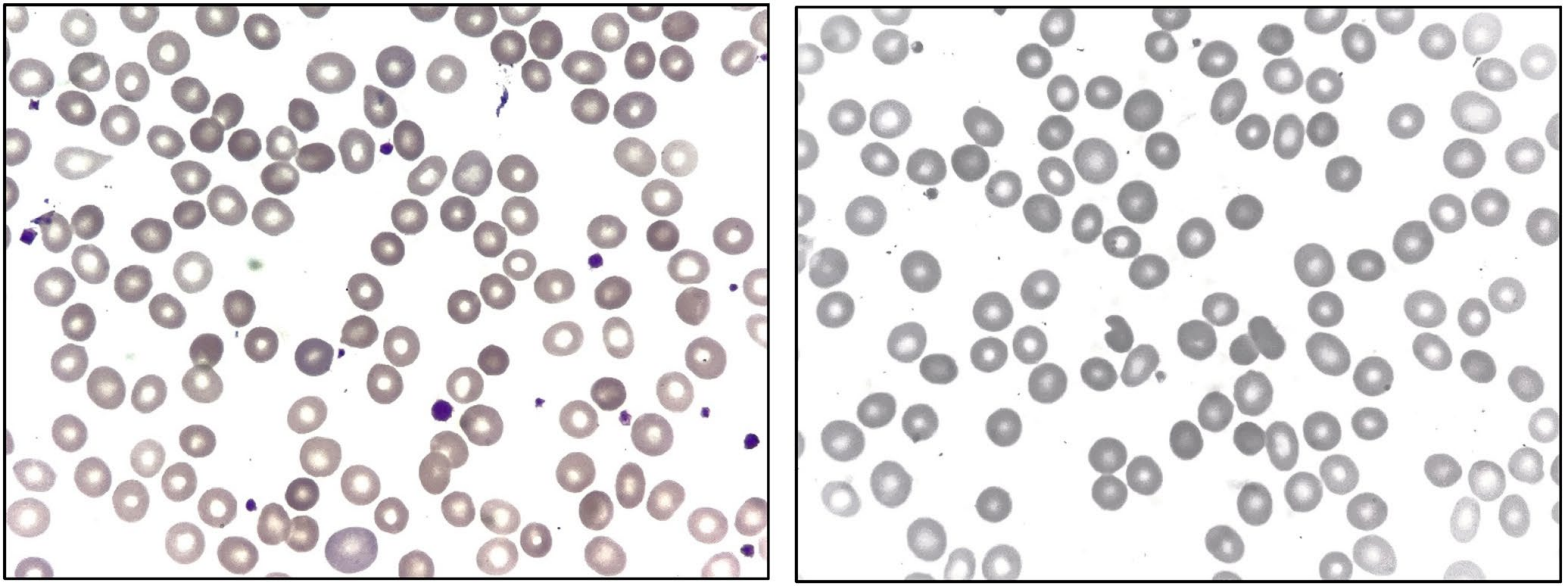


A 50‐year‐old Spanish woman was referred with a diagnosis of hereditary spherocytosis. She had undergone cholecystectomy 8 years earlier and had required blood transfusions during each of her three pregnancies; none of her children was affected. Initial laboratory data showed a hemoglobin concentration of 93 g/L, mean corpuscular volume (MCV) of 95.7 fL, mean corpuscular hemoglobin (MCH) of 33 pg, and mean corpuscular hemoglobin concentration (MCHC) of 354 g/L. The absolute reticulocyte count was elevated at 218 × 10^9^/L. Platelet and leukocyte counts were normal. Her blood film showed moderate numbers of spherocytes and small numbers of other poikilocytes (tear‐drop poikilocytes, bite cells and irregularly contracted cells) (images, May–Grünwald–Giemsa ×100 objective). There was also polychromasia. Total bilirubin was elevated at 2.25 mg/dL, with a direct fraction of 0.48 mg/dL. Haptoglobin was undetectable. Lactate dehydrogenase and liver enzymes were normal. Flow cytometry‐based eosin‐5‐maleimide (EMA) binding assay was repeatedly normal, with a fluorescence ratio of 0.96. Abdominal ultrasonography revealed a large, homogeneous spleen measuring 13.4 cm in its longest diameter.

A next‐generation sequencing (NGS) panel for hereditary hemolytic anemias was analyzed. This showed the nonsense variant c.4519C>T p.(Arg1507*) in the *SPTA1* gene, predicting substitution of an arginine by a premature stop codon at position 1507 [1]. The patient also carried two common variants in *SPTA1—*c.6531‐12C>T and c.5572C>G p.(Leu1858Val). In addition, a heterozygous variant of uncertain significance was detected in the *GSR* gene, which encodes glutathione reductase: c.616A>C p.(Thr206Pro).

Hereditary spherocytosis (HS) comprises a heterogeneous group of red cell membrane disorders, with an estimated prevalence of at least one in 2000 among individuals of Northern European descent. Approximately three‐quarters of cases have an autosomal dominant inheritance pattern, while the remainder are either sporadic de novo mutations or inherited in an autosomal recessive manner [2]. Pathogenic mutation of *SPTA1* is responsible in only a minority of cases, occurring when there is homozygosity, compound heterozygosity, or coinheritance with a low expression allele in *trans*, such as α spectrin^LELY^ (**L**ow **E**xpression **Ly**on) [3] or α spectrin^LEPRA^ (**L**ow **E**xpression **P**rague) [4]. The designation α spectrin^LELY^ has sometimes been used to refer specifically to the c.6531‐12C>T variant but often to designate its coexistence with the c.5572C>G p.(Leu1858Val) variant in *cis*. This polymorphism, as observed in our patient, is common in the general population, with an allele frequency of approximately 20%–30% [4].

EMA binding is often used for confirmation of a diagnosis of hereditary spherocytosis when spherocytes are observed in the blood film of a patient with chronic hemolytic anemia. However, the observation of normal or borderline EMA binding does not exclude this diagnosis, and in this circumstance, detailed genetic analysis is indicated. It is also worth noting that this patient harbored a heterozygous variant in the *GSR* gene, which encodes glutathione reductase. While its pathogenicity is uncertain, impaired glutathione recycling may reduce the erythrocyte's ability to manage oxidative stress, potentially acting as a phenotype modifier in the setting of chronic hemolysis.

## Conflicts of Interest

The authors declare no conflicts of interest.

## Data Availability

The authors have nothing to report.
